# Improved Temporal Response of MoS_2_ Photodetectors by Mild Oxygen Plasma Treatment

**DOI:** 10.3390/nano12081365

**Published:** 2022-04-15

**Authors:** Jitao Li, Jing Bai, Ming Meng, Chunhong Hu, Honglei Yuan, Yan Zhang, Lingling Sun

**Affiliations:** 1 School of Physics and Telecommunications Engineering, Zhoukou Normal University, Zhoukou 466001, China; lijitao@zknu.edu.cn (J.L.); mengmingfly@163.com (M.M.); yhl@zknu.edu.cn (H.Y.); zhangyan@zknu.edu.cn (Y.Z.); 2 The Key Laboratory of Rare Earth Functional Materials of Henan Province, Zhoukou Normal University, Zhoukou 466001, China; 3 Department of Foundation Laboratory, Army Engineering University of PLA, Nanjing 210023, China; dnbaijing@163.com; 4 College of Life Science and Agronomy, Zhoukou Normal University, Zhoukou 466000, China; ourcarrot@163.com

**Keywords:** MoS_2_ photodetector, trap states, temporal response, oxygen plasma treatment

## Abstract

Temporal response is an important factor limiting the performance of two-dimensional (2D) material photodetectors. The deep trap states caused by intrinsic defects are the main factor to prolong the response time. In this work, it is demonstrated that the trap states in 2D molybdenum disulfide (MoS_2_) can be efficiently modulated by defect engineering through mild oxygen plasma treatment. The response time of the few-layer MoS_2_ photodetector is accelerated by 2–3 orders of magnitude, which is mainly attributed to the deep trap states that can be easily filled when O_2_ or oxygen ions are chemically bonded with MoS_2_ at sulfur vacancies (SV) sites. We characterized the defect engineering of plasma-exposed MoS_2_ by Raman, PL and electric properties. Under the optimal processing conditions of 30 W, 50 Pa and 30 s, we found 30-fold enhancements in photoluminescence (PL) intensity and a nearly 2-fold enhancement in carrier field-effect mobility, while the rise and fall response times reached 110 ms and 55 ms, respectively, at the illumination wavelength of 532 nm. This work would, therefore, offer a practical route to improve the performance of 2D dichalcogenide-based devices for future consideration in optoelectronics research.

## 1. Introduction

Molybdenum disulfide (MoS_2_) is a representative of transition metal dichalcogenides (TMDCs) material with easy preparation via CVD and/or exfoliation, high electron mobility (>100 cm^2^/Vs), and an abundant form of MoS_2_ as natural minerals [1,2]. When MoS_2_ flakes are scaled down to the monolayer, a transition from indirect to direct bandgap occurs due to the quantum confinement effect [3]. Recently, optoelectronic devices fabricated from MoS_2_ have received notable attention. The MoS_2_-based photodetectors have reported remarkable performances including broadband detection from ultraviolet to near-infrared (NIR) [4], ultra-high photoresponsivity [5], flexible application [6], polarization sensitive photodetection [7] and so on. However, most of the studies have focused on the photoresponsivity of the MoS_2_ photodetector, such as back-gating [8], evaporation of sub-stoichiometric molybdenum oxide overlayers [9] and surface sensitization using PbS and MoO quantum dots [10,11], organic molecules [12], photogating effects [13] and metal nanostructures [14]. In order to realize the real application of MoS_2_-based photodetectors in imaging, display and other fields, the improvement of temporal response is crucial.

The trap states in semiconductor photoconductors play important roles in prolonging the lifetime of photoexcited carriers and hence strongly influence the response or decay time of the photoconductor [15,16]. Due to the ultrathin thickness of 2D materials, the electronic properties are more easily modulated through defect and interface engineering [17,18], which are commonly used methods to control the trap states. For example, Jiang et al. modulated the trap states in a ReS_2_ nanosheet by surface adsorption of organic molecules, thus accelerating the response time of the photodetector by 3–4 orders of magnitude [19]. Shim et al. created the trap states on the surface of the ReS_2_ by O_2_ plasma thinning, resulting in a photodetector with a fast response time [20]. By alloying MoS_2_ with Sn during CVD growth, Mo et al. introduced defect states in the monolayer MoS_2_ and accelerated the response time of the photodetector to 20 ms (rising) and 26 ms (decaying) [21]. By inserting an atomic layer deposited TiO_2_ film between the sample and substrate, Yusin Pak et al. effectively enhanced the response speed of MoS_2_ devices [22]. However, these methods often involve additional preparation steps or are prone to damage/thinning of the sample.

The plasma technique has been proposed as a feasible means of modulating the properties in 2D materials [23]. In particular, oxygen-containing plasmas tend to form oxide centers on the surface of MoS_2_, and these oxide centers can then act as dopants that alter the charge concentration in the modified MoS_2_ transistor channel, and ultimately improving the photoresponsivity [24]. The specific ratio of oxygen/argon plasma can be precisely controlled and improve the mobility and conductivity of few layer MoS_2_, which is mainly due to the modulation of electronic behavior by a transient 2D substoichiometric phase of molybdenum trioxide (2D-MoO*_x_*) [25]. In this work, we demonstrate that the temporal response of MoS_2_ based photodetectors can be accelerated by mild O_2_ plasma treatment. Unlike other reported plasma treatment methods, our soft plasma works in the capacitive discharge mode (E-mode), where the inductively-coupled electric field originated from the mutual induction between the coil and the plasma is much smaller than the radial electrostatic field induced by the radial potential drop across the two ends of the planar induction coil. As such, the ion density was also too low to induce destructive ion bombardment onto the processed samples. The response time of the plasma treated MoS_2_ is 2–3 orders faster. At the same time, the field-effect mobility of the device under treatment improves by 2-fold of magnitude, and the photoluminescence intensity has been enhanced by nearly 30 times. Raman spectroscopy, as well as output and transfer curves, were used to characterize the sample after plasma exposure and combined with band theory, we attribute the observed improvement in response time to the chemical bonding of O_2_ or oxygen ions to MoS_2_ on the SV site, thus filling the deep trap states. This work realizes the simultaneous improvement of MoS_2_ PL and device response speed and provides a new solution for the application of 2D materials in optoelectronic devices.

## 2. Materials and Methods

The MoS_2_ nanosheets were mechanically exfoliated from highly oriented pyrolytic crystal (SPI Supplies, West Chester, PA, USA) and deposited onto a lightly p-doped silicon substrate that is terminated with 300 nm of SiO_2_. Raman spectra were measured at room temperature using Renishaw Invia micro (Gloucestershire, UK) with a laser excitation of 532 nm.

Photodetector devices were fabricated using standard electron beam lithography (FEI, FP2031/12 INSPECT F50, Hillsboro, FL, USA) followed by thermal evaporation (TPRE-Z20-IV, Changchun, China) of 5 nm Ni and 50 nm Au. A planar medium-frequency (2 MHz) inductively coupled plasma (ICP) source was applied to treat the MoS_2_ nanosheets at room temperature. Electrical and photoresponse characteristics of the devices were measured using a Keithley 2634 analyzer (Cleveland, OH, USA) under ambient conditions and a 532 nm laser was used as a light source.

## 3. Results

**Figure 1:**
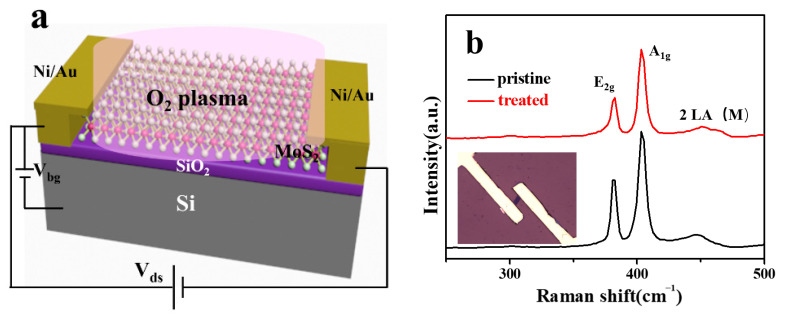

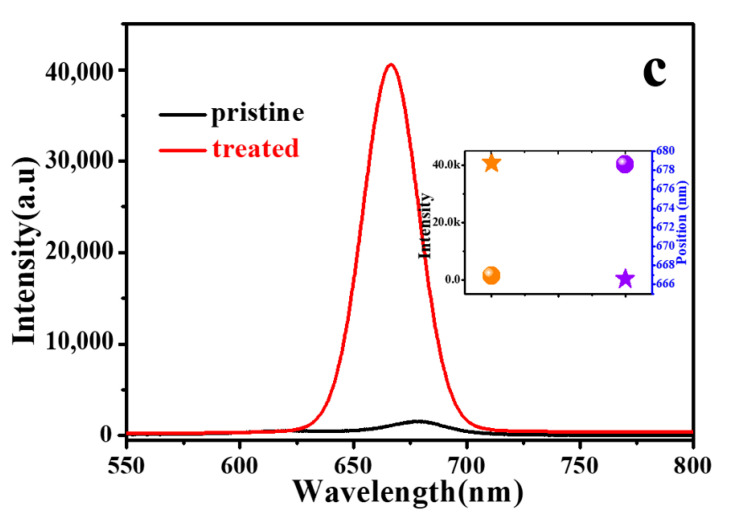
Evolution of optical properties of MoS_2_ after mild oxygen plasma irradiation. (**a**) Schematic of the process of mild plasma treatment of MoS_2_ device. (**b**) Raman and (**c**) PL spectra of few layer MoS_2_ before and after oxygen plasma treatment. Inset of (**b**) is the image of the MoS_2_ device and inset of (**c**) is the intensity and position of PL peaks.

Figure 1a shows the schematic of the process of mild O_2_ plasma treatment of the MoS_2_ device, and the image of the device is shown in the inset of Figure 1b. In order to ensure a better light absorption, the thickness of MoS_2_ was selected as 9.8 nm (shown in Figure S1), and the corresponding bandgap is 1.3 eV. Meanwhile, in order to ensure the timeliness of the treatment effect, plasma treatment was carried out after the device was completed, and the treatment conditions were 30 W, 50 Pa and 30 s. The Raman spectra before and after plasma treatment are plotted in Figure 1b, the observed typical peaks at 383 cm^−1^ and 408 cm^−1^ can be attributed to the in-plane E^1^_2g_ phonon mode and the out-of-plane A_1g_ mode [26]. Through the fitting analysis of peak position and intensity, it can be found that compared with the pristine sample, the E^1^_2g_ peak of the treated sample was decreased and slightly broadened. At the same time, the A_1g_ peak also undergoes a slight blue shift. The variation of the Raman spectrum indicated that p-type doping of the MoS_2_ sample occurs during the oxygen plasma treatment process [27]. Of course, the photoluminescence (PL) intensity is more sensitive to doping effects. As shown in Figure 1c, the PL intensity of the MoS_2_ flake can be strongly enhanced by 30 times after the oxygen plasma treatment, along with a ~12 nm blue shift. The as-prepared few layer MoS_2_ is normally n-doped, due to the presence of sulfur vacancies (SVs) on the surface in intrinsic MoS_2_, which will act as electron donors and induce localized states in the bandgap [28]. In such a case, the photoexcited electron-hole pairs will combine with the excess electrons to form negative trions, resulting in fluorescence peaks located at smaller energies. After the oxygen plasma exposure, on one hand, the adsorption of O_2_ molecules or atoms will consume the excess electrons, thus switching the PL process from trion recombination to exciton recombination. On the other hand, the dangling bond density on the surface will be reduced, which was mainly caused by deep level trap states in bandgap formed by defects, and thus the radiative recombination rate increases considerably, resulting in a dramatic increase in PL.

**Figure 2:**
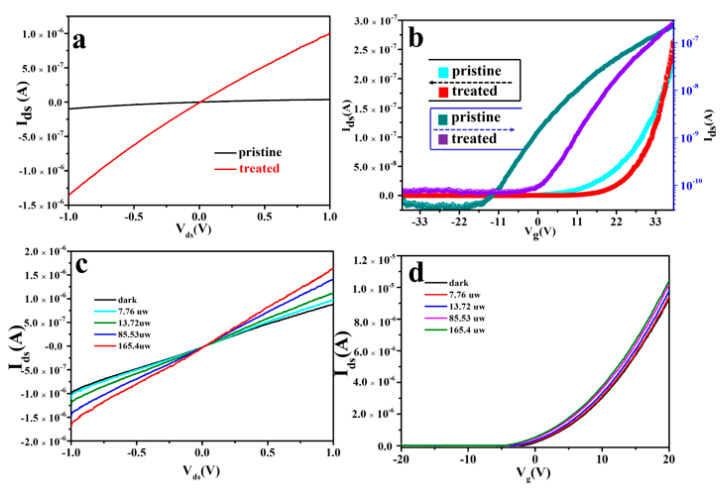
Evolution of electric properties of MoS_2_ after mild oxygen plasma irradiations. (**a**) Out curves and (**b**) transfer curves of few layer MoS_2_ before and after oxygen plasma treatment. (**c**) The dependence of the *I*_ds_-*V*_ds_ curves of the post-plasma treatment MoS_2_ on the laser power. The wavelength of laser is 532 nm. *I*_ds_ increasing with the laser power. (**d**) Transfer characteristics of the treated device, small changes indicate shielding of photo-grating effects.

Next, we investigated the effects of mild oxygen plasma treatment on the electrical performance of MoS_2_ flakes. The typical output and transfer curves of oxygen plasma modified and pristine devices are shown in Figure 2a,b. It can be seen from Figure 2a, that the output characteristic curves are indeed asymmetric, and these absences of ambipolarity are mainly attributed to the suppression of hole current by the Schottky barriers. It is well known that, according to the Schottky Mott theory, the expected barrier height should be close to zero for a defect-free Ni/MoS_2_ interface. However, in novel materials, such as MoS_2_ channel devices, the interface between the metal and semiconductor always can be affected by many factors, such as surface adsorbates, defects, etc., which will lead to the formation of the Fermi pinning effect, resulting in the formation of the Schottky Barrier. Meanwhile, the electron conductance of the MoS_2_ device became better. According to the formula of electron conductance: *G* = *I*_ds_/*V*_ds_, it can be concluded that the conductivity increased by 10 times, that is, from 0.13 μs to 1.37 μs. This is mainly because, during the oxygen plasma treatment, oxygen molecules have a certain probability to intercalate into the interlayers of MoS_2_ layers, thereby forming a MoS_2_[O_2_]*_x_* superlattice structure [29,30]. In this way, the conductive channel will change from the original MoS_2_ flakes to the parallel connection of MoS_2_ and MoS_2_[O_2_]*_x_*, resulting in a smaller resistance. However, it is worth noting that although the existence of the superlattice can be confirmed by the decomposition results of the oxygen bond spectrum in XPS (shown in Figure S5), it can be seen from the following experimental results that the main effect of oxygen plasma treatment is to repair defects by doping. Moreover, the specific probability of intercalation caused by the process of oxygen plasma treatment is worth further study. For the transfer characteristic curve of Figure 2b, the bias voltage is fixed at 0.1 V, and the left ordinate in the figure is in linear form, while the right ordinate is in exponential form. The field-effect mobility was calculated using the following equation:

(1) $$\mu =\frac{L}{W\times (\varepsilon_{0}\varepsilon_{r}/d)\times V_{\mathrm{ds}}}\times \frac{dI_{\mathrm{ds}}}{dV_{g}}$$

where *L* is the channel length, *W* is the channel width, *ε*_0_ and *ε*_r_ are the absolute and relative dielectric constant, respectively, and d is the thickness of SiO_2_ (300 nm). After mild oxygen plasma treatment, the electron mobility is changed from ~7.61 to ~14.4 cm^2^ V^−1^ s^−1^. These results clearly demonstrate that the electrical performance of MoS_2_ could be greatly improved by mild oxygen plasma treatment. It has been reported that for the pristine MoS_2_, the point defects, e.g., SVs would lead to the formation of localized states in the bandgap, and result in hopping transport behavior [28]. When O_2_ or oxygen ions are bonded with MoS_2_ at SV sites, the localized defect states can be easily fulfilled and scattering centers are removed, increasing mobility [31]. In conclusion, the increase in conductivity and mobility of MoS_2_ after oxygen plasma treatment is ultimately due to the reduction of trap states caused by defects.

Figure 2c shows the output characteristics of the device after plasma treatment under laser illumination with 532 nm. The transistor channel length was smaller than the area of the laser spot, confining the laser irradiation solely to the MoS_2_ region. As the laser power increases, the photocurrent gradually increases, which is typical for semiconducting devices. Moreover, the current reaches 1.5 μA at ± 1 V under the laser power of 165.4 μW. Compared with the untreated sample (shown in Figure S2), the current increases by a large magnitude under the same conditions. This indicates that dopants introduced by the plasma treatment to the MoS_2_ surface mediate an enhanced photo-generation response of the charge carrier in the device. Similar to the output curve, the transfer characteristic curve (Figure 2d) also increases with the laser power, but the change is very small, indicating that the doping of oxygen plasma effectively shields the photo-grating effect in MoS_2_. This photo-grating effect in low-dimensional photodetectors can be simply ascribed to the prolonged excess carrier lifetime induced by defects and impurities [16].

**Figure 3:**
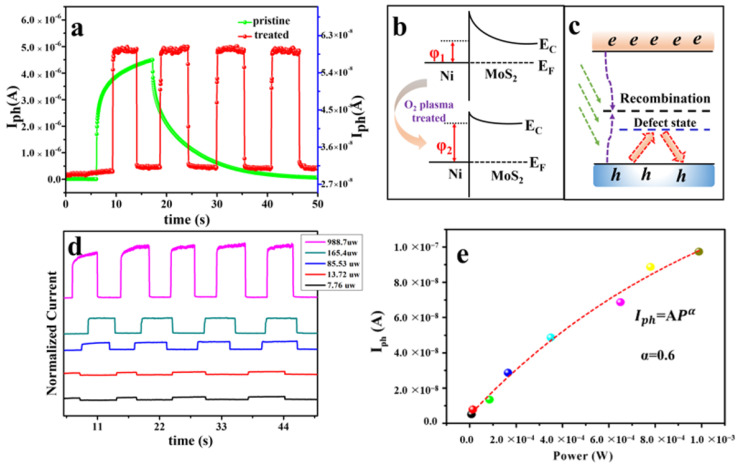
(**a**) Transient response of as-prepared and plasma treated MoS_2_. (**b**) Energy band diagrams of Ni/MoS_2_ junctions before (top) and after (bottom) O_2_ plasma treatment of few layer MoS_2_. (**c**) Schematic diagram of the band structure of the treated MoS_2_, different to pristine MoS_2_, the deep trap states are filled with oxygen molecules, only leaving shallow defect traps. (**d**) The temporal time-dependent light current (*I*_ph_) under laser illumination with a different laser power. (**e**) The relationship between the *I*_ph_ and the laser power, and it can be fitted by *I*_ph_ = *AP^α^*, where *α* is estimated to be 0.6.

The transient response of as-prepared and plasma treated devices is shown in Figure 3a. According to the definition of response time, which is the time between 90% and 10% of the maximum value of the current, it can be found that the rise time and fall time of pristine MoS_2_ photodetector are 10 s and 31 s, respectively. The timescale of minutes of response time is consistent with previous reports [24]. It is well known that for 2D materials prepared by mechanical exfoliation, defects are inevitably formed in the samples due to stress, surface adsorption and so on. In the presence of a large number of defects traps, the quasi Fermi level for holes will be pinned even under high illumination intensity. This is the reason why defects play a dominant role in as-prepared MoS_2_ photoconductors. On the other hand, the response time of treated samples is nearly three orders of magnitude faster, with a rise of 110 ms and 55 ms, respectively. However, it is worth noting that there is a slight decrease in the photocurrent of the MoS_2_ photodetector after oxygen plasma treatment. According to the schematic description in Figure 3b, the decrease of photocurrent seems to be attributed to the p-doping phenomenon based on surface oxidation, which allows the quasi-Fermi level of holes to shift down. This consequently increases the height of the potential barrier at the Ni/MoS_2_ junction interface, thereby inhibiting the injection of electron carriers from Ni to MoS_2_. Moreover, the improvement in the response rate is mainly due to the modulation of the trap states caused by the defects. In the process of oxygen plasma treatment, oxygen molecules will gradually fill the defects in MoS_2_. According to previous literature reports, oxygen molecules will first bond with the vacancy causing deep level defects to form localized defect states. As a result, the shallow level traps are in a dominant position while the contribution from deep level traps becomes negligible. As shown in Figure 3c, when deep level defects are filled, the recombination and separation rates of photogenerated carriers will rapidly increase under illumination, thus improving the response speed. This is consistent with the mechanism of PL enhancement in Figure 1c. Nevertheless, the response time we achieved here is still far from practical applications and can be further reduced by constructing p-n/Schottky junction detectors.

The detection performance under different incident laser powers (7.76, 13.72, 85.53, 165.4, and 988.7 μW) is shown in Figure 3d. The light current (*I*_ill_) displays a monotonous increase with the increase of incident laser power. The plots of photocurrent (*I*_ph_) as a function of the incident laser power are shown in Figure 3e. The *I*_ph_ and *R* are calculated according to the following equations:

(2) $$I_{\mathrm{ph}}=I_{\mathrm{ill}}-I_{\mathrm{dark}}$$

(3) $$R=I_{\mathrm{ph}}/P$$

where *I*_ill_, *I*_dark_, and P represent the light current, dark current, and incident power, respectively. The photocurrent exhibits a monotonic increase with the incident laser power while the photoresponsivity reaches a maximum value of 0.65 mA/W under the minimum incident laser power of 7.76 μW. Furthermore, the dependences of *I*_ph_ on the incident laser power can be fitted by *I*_ph_ = *AP^α^*, where *α* is estimated to be 0.6. While, the *α* of the pristine sample is about 0.25 (Figure S3), and the increase of the value of *α* indicates that the defect content in the sample decreases, but some defects are still not completely repaired. This is similar to that reported in the previous [31].

**Figure 4:**
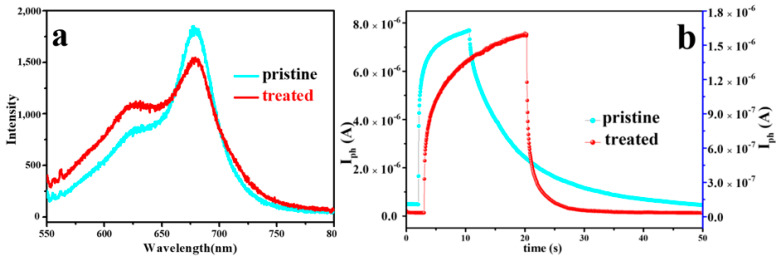
Evolution of PL spectra (**a**) and transient response (**b**) of MoS_2_ before and after treatment under mild oxygen plasma conditions of 10 W, 50 Pa, 10 s.

Until now, we have demonstrated that oxygen plasma treatment under suitable conditions can effectively localize the deep level defects in the intrinsic MoS_2_ nanosheet, thus increasing the PL intensity and accelerating the photocurrent response. To further verify the results, we changed the plasma treatment conditions (10 W, 50 Pa, 10 s) and treated another MoS_2_ device with the same thickness, the results are shown in Figure 4a, it can be seen that the PL intensity of MoS_2_ after treatment under this condition did not increase but slightly decrease, which indicated that the oxygen molecules have not bonded with the vacancies in the samples, but simply oxidized the surface samples [24], and this oxidation was mainly due to the fact that more S atoms were taken away during the plasma treatment, thus increasing the probability of the presence of oxygen atoms or molecules. At the same time, we monitored the photoelectric response of this sample and found that neither the responsivity nor the response time changed significantly (shown in Figure 4b). Compared with the response speed of the previous device with greatly enhanced PL intensity, the performance of this MoS_2_ photodetector more intuitively demonstrates that the repair of deep-level defects traps by oxygen plasma treatment is the main reason for improving the response speed of the MoS_2_ photodetector. On the other hand, this further demonstrates the advantages of plasma treatment for modulating the performance of 2D materials, and different effects can be achieved by changing the treatment conditions.

## 4. Conclusions

In conclusion, we have demonstrated that the temporal response of the MoS_2_ photoconductor can be improved by 2–3 orders after oxygen plasma treatment. This is mainly due to the deep trap states can be easily filled when O_2_ or oxygen ions are chemically bonded with MoS_2_ at SV sites. These deep trap states are the main factor for prolonging the photoresponse time of 2D materials. Our results reported here could provide a valuable route for fabricating high performance 2D nanodevices for electronic and optoelectronic applications.

## Data Availability

The data is available on reasonable request from the corresponding author.

## Supplementary Materials

The following supporting information can be downloaded at: https://www.mdpi.com/article/10.3390/nano12081365/s1, Figure S1: AFM surface morphology of the pristine MoS_2_ nanosheet. Figure S2: The dependence of the *I*_ds_-*V*_ds_ curves of the pristine MoS_2_ on the laser power. Figure S3: The relationship between the *I*_ph_ and the laser power of the pristine MoS_2_ photodetector. Figure S4: XPS spectra of Mo 3d and S 2s core levels of (a) as-prepared and (b) oxygen-plasma treated MoS_2_. Figure S5: Transfer characteristics of the treated device in a semi-logarithmic coordinate. Figure S6: XPS spectra of O 1s core levels of pristine and oxygen-plasma treated MoS_2_. Figure S7: Schematic of MoS_2_[O_2_]*_x_* superlattices structure.
