# Supplementary material for: Improved Temporal Response of MoS2 Photodetectors by Mild Oxygen Plasma Treatment
Source: Nanomaterials (Basel). 2022 Apr 15;12(8):1365. doi: 10.3390/nano12081365 (PMC9031829; doi:10.3390/nano12081365)
Supplement: Supplementary file 1 [file nanomaterials-12-01365-s001.zip › nanomaterials-1615548-supplementary.pdf]

Supplementary Materials

Improved Temporal Response of MoS_2 Photodetectors by Mild Oxygen Plasma Treatment

Jitao Li [1,2], Jing Bai [3], Ming Meng [1], Chunhong Hu [4], Honglei Yuan [1], Yan Zhang [1] and Lingling Sun [1,*]

1 School of Physics and Telecommunications Engineering, Zhoukou Normal University,
Zhoukou 466001, China; lijitao@zknu.edu.cn (J.L.); mengmingfly@163.com (M.M.); yhl@zknu.edu.cn (H.Y.); zhangyan@zknu.edu.cn (Y.Z.)
2 The Key Laboratory of Rare Earth Functional Materials of Henan Province, Zhoukou Normal University, Zhoukou 466001, China
3 Department of Foundation Laboratory, Army Engineering University of PLA, Nanjing 210023, China; dnbaijing@163.com
4 College of Life Science and Agronomy, Zhoukou Normal University, Zhoukou 466000, China;
ourcarrot@163.com
* Correspondence: sunlingling@zknu.edu.cn; Tel.: +86-1394-8178-990

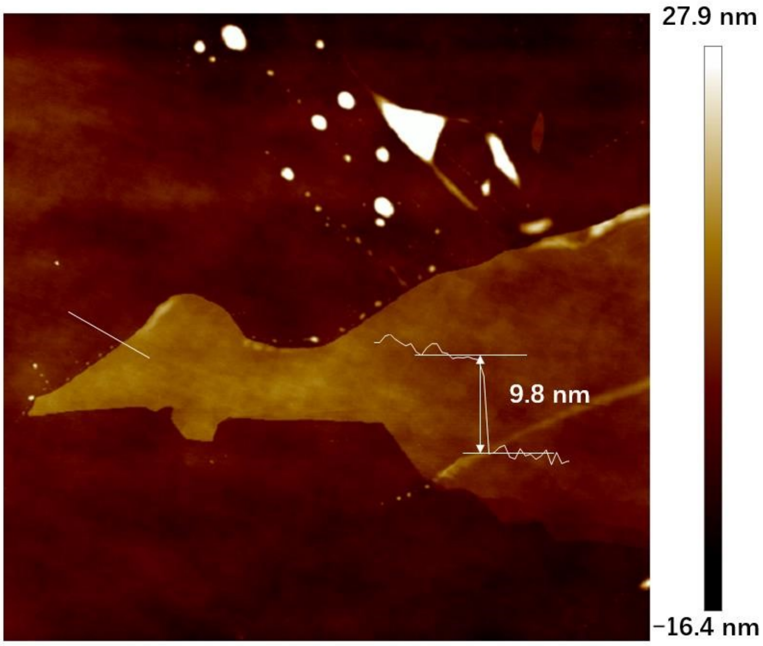

Figure S1. AFM surface morphology of the pristine MoS_2 nanosheet. It can be seen that the thickness of this MoS_2 nanosheet we used is about 9.8 nm.

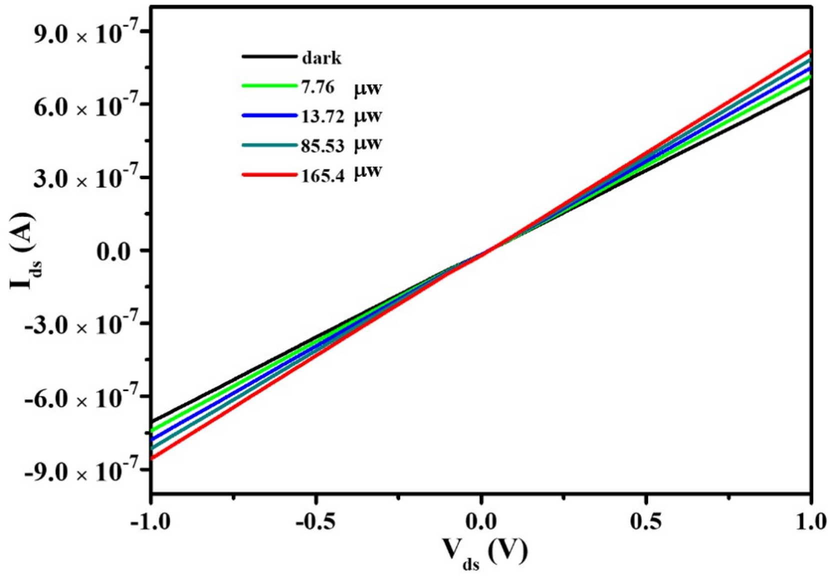

Figure S2. The dependence of the I_{ds}-V_{ds} curves of the pristine MoS_2 on the laser power. The wavelength of laser is 532 nm. It can be seen that I_{ds} increasing with the laser power, but the current only reaches to 0.85 μA at ±1V under the laser power of 165.4 μW, which is much smaller than that the plasma treated sample.

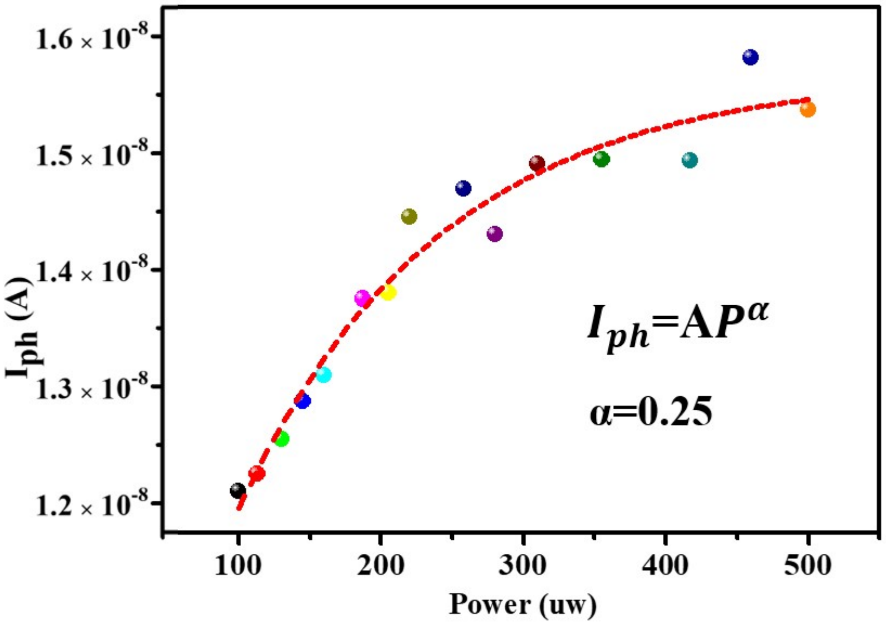

Figure S3. The relationship between the I_{ph} and the laser power of the pristine MoS_2 photodetector. When fitted it with equation:$I_{ph} = AP^{\alpha}$, the α is estimated to be only 0.25, indicating that the concentration of defects in pristine sample is very high.

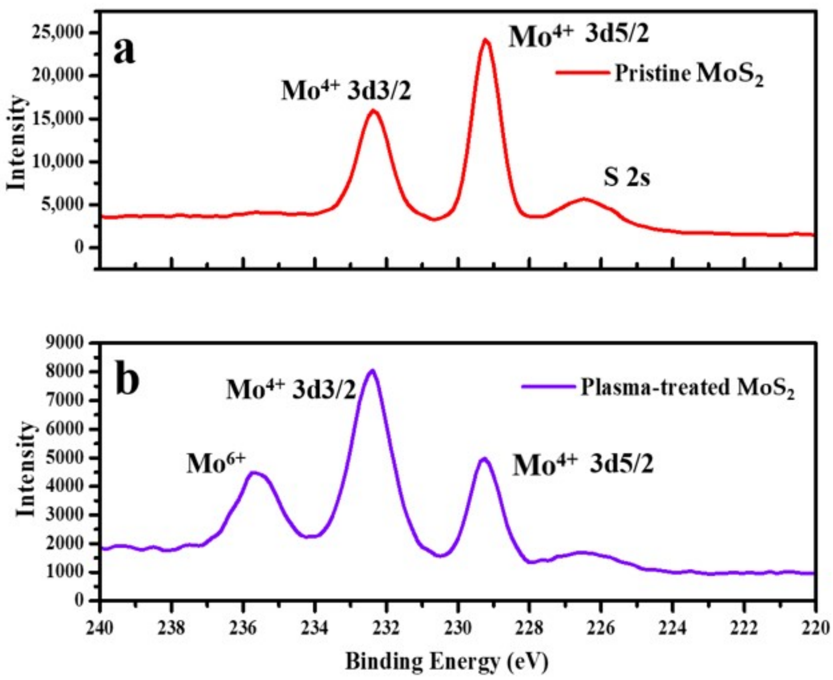

Figure S4: XPS spectra of Mo 3d and S 2s core levels of (a) as-prepared and (b) oxygen-plasma treated MoS_2.

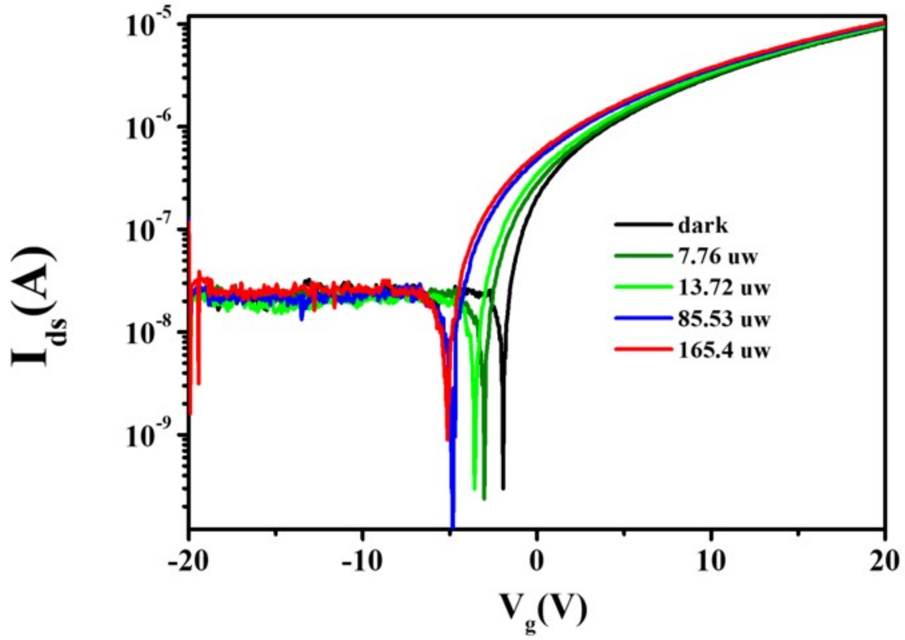

Figure S5: Transfer characteristics of the treated device in a semi-logarithmic coordinate.

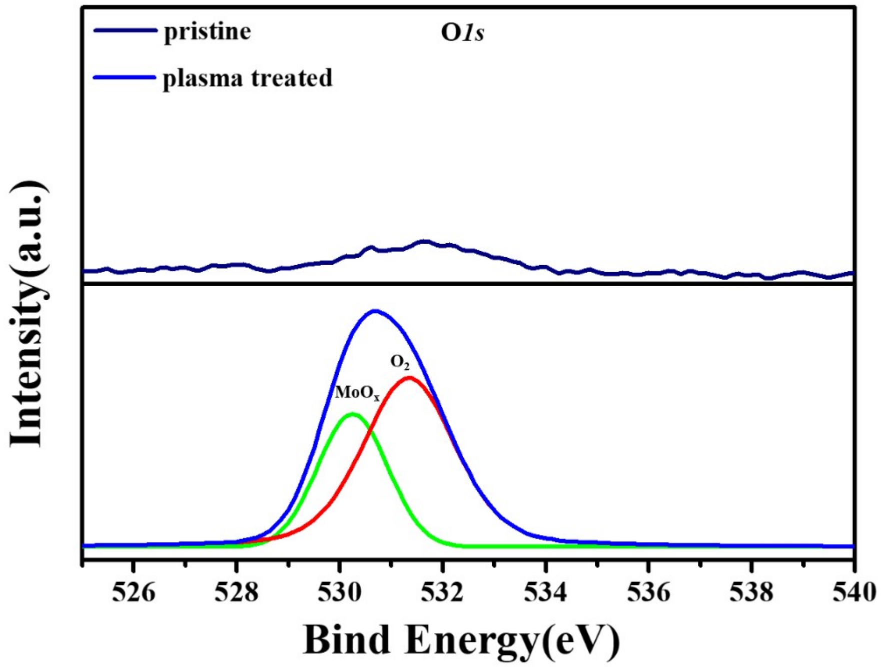

Figure S6: High-resolution XPS spectra of O 1s of the pristine and treated MoS_2 nanosheet. The enhanced O 1s peak can be decomposed into two peaks, one at 530.7 eV and the other at 531.9 eV, representing MoO_x and oxygen molecules, respectively.

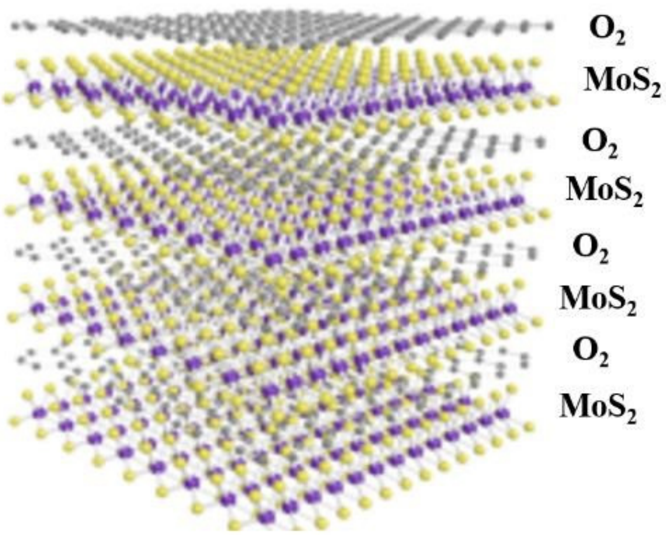

Figure S7: Schematic of $MoS_2[O_2]_x$ superlattices structure
